# Approaches to denote treatment outcome: Clinical significance and clinical global impression compared

**DOI:** 10.1002/mpr.1797

**Published:** 2019-10-09

**Authors:** Edwin de Beurs, Ingrid V.E. Carlier, Albert M. van Hemert

**Affiliations:** ^1^ Section Clinical Psychology Leiden University Leiden The Netherlands; ^2^ Department of Psychiatry Leiden University Medical Center Leiden The Netherlands

**Keywords:** clinical global impression, clinical significance, Reliable Change Index, treatment outcome

## Abstract

**Objectives:**

The authors of a previous study proposed a statistically based approach to denote treatment outcome, translating pretest and posttest scores into clinically relevant categories, such as recovery and reliable improvement. We assessed the convergent validity of the Jacobson–Truax (JT) approach, using T‐score based cutoff values, with ratings by an independent evaluator.

**Methods:**

Pretest and retest scores on the Brief Symptom Inventory (BSI) and clinical global impression improvement (CGI‐I) ratings were collected repeatedly through routine outcome monitoring from 5,900 outpatients with common mental disorders. Data were collected in everyday practice in a large mental health care provider.

**Results:**

Continuous pretest‐to‐retest BSI change scores had a stronger association with CGI‐I than the categorical variable based on JT. However, JT categorization and improvement according to CGI converged substantially with association indices (Somers' *D*) ranging from *D* = .50 to .56. Discordance was predominantly due to a more positive outcome according to JT than on CGI‐I ratings.

**Conclusion:**

Converting continuous outcome variables into clinically meaningful categories comes at the price of somewhat diminished concurrent validity with CGI‐I. Nevertheless, support was found for the proposed threshold values for reliable change and recovery, and the outcome denoted in these terms corresponded with CGI improvement for most patients.

## 1 INTRODUCTION

Whether patients benefit from treatment in clinical practice and in outcome research is usually assessed by repeated measurement of symptoms, functioning, and/or quality of life with reliable and valid self‐report questionnaires, such as the Brief Symptom Inventory (BSI; Derogatis, 1975a), or clinical rating scales, such as the Hamilton Depression Rating Scale (Hamilton, 1960). Repeated assessments may yield a number of indices for outcome, such as a posttest score (end state), a change score (the change in severity from baseline to reassessment), or a residual change score (the change corrected for pretest severity). Each of these indices provides information on outcome with an abstract number, not directly revealing what has been achieved with a patient in clinically meaningful terms, such as recovery, improvement, or deterioration. For instance, a pretest‐to‐posttest shift in score on a measurement scale does not immediately reveal whether the treatment was successful, whereas a change from dysfunctional to functional (i.e., recovery) does reveal its importance and the clinical relevance of what has been achieved. Therefore, in addition to more traditional outcomes, such as the effect size of between group differences (Cohen, 1988) or the size of within‐group or individual pretest‐to‐posttest change (Seidel, Miller, & Chow, 2013), the effectiveness of treatment should also be expressed in outcomes that have real life meaning, both at the individual patient level and at group level when aggregated data are used.

A well‐established method to translate measurements of outcome into clinical meaningful terms is the Jacobson–Truax (JT) approach to clinical significant change (Jacobson, Follette, & Revenstorf, 1986; Jacobson, Roberts, Berns, & McGlinchey, 1999; Jacobson & Truax, 1991). They proposed two indices: clinical significant change and statistically reliable change. Clinical significance (JT_CS_) requires crossing a cutoff value distinguishing the dysfunctional from the functional population. They proposed several cutoff values: two standard deviations below the dysfunctional mean, two standard deviations above the functional mean or the point where the frequency distribution of scores of the functional and dysfunctional population cross lines. When normative data of the dysfunctional and the functional population are available, the last operationalization is preferred.^1^ However, for patients with a pretest score close to the threshold value, a tiny change may be sufficient to cross the cutoff point. Therefore, they proposed the additional criterion of the Reliable Change Index (JT_RCI_). This is the amount of change required to be 95% certain that change is statistically reliable and not due to measurement error of the instrument used. Combining both indices results in five categories: recovered (reliably improved and changed from dysfunctional to functional), reliably improved (improved, but still dysfunctional), unchanged (not reliably changed), reliably deteriorated (reliably changed towards worsening of the condition), and relapsed (reliably deteriorated and changed, but now from functional to dysfunctional). The last category is usually small (few patients score at baseline as functional) and is sometimes merged with reliable deterioration.

Several cutoff values are required to categorize patients into the five categories of JT: (1) a positive and negative value for statistical reliable change to distinguish improved and deteriorated patients from unchanged patients (JT_RCI_) and (2) a value to distinguish clinical significantly changed (or recovered) patients from merely improved patients (JT_CS_). Previously, we proposed generic cutoff values for these indices to be used when raw scores on measures have been transformed to T‐scores: JT_RCI_ > 5.0 (or JT_RCI_ < −5.0) and JT_CS_ = 55 (de Beurs et al., 2016; de Beurs, Flens, & Williams, 2019). These values are based on the formulas provided by Jacobson et al. (1999) and a reliability coefficient for the BSI total score (BSI‐TOT) of Cronbach's *α* = .97 and means M_T_ = 60 and M_T_ = 50 for patients and the general population, respectively.^2^ It has become increasingly common to score measurement instruments on the generic T‐score scale (Cella et al., 2010; Kaat, Newcomb, Ryan, & Mustanski, 2017; Wahl et al., 2014). The proposed threshold values coincide with minimally detectable change of half a standard deviation (Norman, Sloan, & Wyrwich, 2003) and with the value proposed by the Patient‐reported Outcomes Measurement Information System (PROMIS) initiative (see www.healthmeasures.net/explore-measurement-systems/promis) to distinguish a T‐score within normal limits from a mild level of problems (*T* > 55). Other categories for T‐scores proposed by PROMIS are mild (*T* = 55 to 60), moderate (*T* = 61 to 70), and severe (*T* > 70).

The JT approach is well established, firmly based on psychometric and statistical considerations, and recommended to be included in all psychotherapy outcome studies (Lambert & Ogles, 2009; Nezu & Nezu, 2007). However, it has been criticized as well on various grounds (Kazdin, 1999; Wise, 2004). First of all, the JT_RCI_ criterion can yield a quite conservative indicator of change (Bullinger et al., 1998; Cella, Bullinger, Scott, & Barofsky, 2002; Eisen, Ranganathan, Seal, & Spiro, 2007), as the RCI requires outcome measurement instruments with a high reliability and precision (e.g., Cronbach's *α* > .90, SE_T‐score_ < 3,16). Furthermore, the method requires normative data for the outcome instrument used, preferably from dysfunctional and from functional or general population samples. Also, the method is of limited use with patients who enter treatment scoring in the functional range (Lambert & Ogles, 2009). Finally, Lunnen and Ogles (1998) argue that the approach fails in identifying deterioration, as it distinguishes insufficiently between unchanged and deteriorated cases, again due to the requirement of high measurement reliability and precision. Also, studies use different means, standard deviations, and reliability indicators to calculate cutoff values, even when the same outcome instrument is used (Lambert & Ogles, 2009). This hampers comparison of results across studies.

An empirical evaluation of the JT approach and the proposed cutoff values using T‐scores vis‐à‐vis an external criterion of therapeutic response is in order. Are these cutoff values well chosen and appropriate? Do patients, categorized according to the JT criteria in various groups, differ from each other according to other outcome criteria? A suitable external validation criterion might be found in ratings of the clinical severity and treatment outcome of patients by independent evaluators. In a large routine outcome monitoring (ROM) project (de Beurs et al., 2011), we collected such ratings from extensively trained research nurses who assessed the patients and completed the Clinical Global Impression scale (CGI; Guy, 1976). The validity of the CGI is supported in many studies (Beneke & Rasmus, 1992; Haro et al., 2003; Kadouri, Corruble, & Falissard, 2007; Khan, Khan, Shankles, & Polissar, 2002; Leucht & Engel, 2005; Zaider, Heimberg, Fresco, Schneier, & Liebowitz, 2003). However, as patients' self‐reports and ratings by independent observers stem from different sources, both may hold divergent views on what has been achieved in therapy. For instance, Forkmann et al. (2011) compared staff ratings on the CGI with patients self‐reports on the Beck Depression Inventory (Beck & Steer, 1987) and found only moderate correspondence between both viewpoints. The present study reports on the concordance between the JT categorization, as applied to repeated assessments with the BSI, and ratings of severity and outcome by research nurses on the CGI. Thus, the validity of the JT approach is investigated by comparing it with the clinical judgement of experienced independent raters, and the sensitivity and specificity of proposed cutoff values for CGI‐improvement categories are determined.

## 2 METHODS

### 2.1 Participants

A total of 5,900 outpatients were included (3,704 females, 62,8%; age *M* = 40.0 years; *SD* = 13.7), all referred to GGZ Rivierduinen (a large mental health care provider in an area with 1.1 million inhabitants). According to a semistructured diagnostic interview, the Mini‐International Neuropsychiatric Interview (MINI‐plus; Sheehan et al., 1998), most patients suffered from a singular anxiety (27,9%), singular mood (24,8%), or a comorbid mood and anxiety disorder (26,4%). The remaining 20.9% suffered from other mental disorders (predominantly somatoform disorders) or did not meet *Diagnostic and Statistical Manual of Mental Disorders* criteria. Patients were treated according to evidence‐based guidelines with a combination of pharmacological and psychological treatments. From a related study in GGZ Rivierduinen, we know that major depression disorder is more frequently treated with pharmacotherapy than psychotherapy (55% and 24%, respectively), and this is the reverse for anxiety disorders (23% and 59%). For both conditions, the remaining minority is treated with combinations or with other treatments (van Fenema, van der Wee, Giltay, den Hollander‐Gijsman, & Zitman, 2012). Guideline adherence in general was good.

The Medical Ethical Committee of the Leiden University Medical Center approved the general study protocol regarding ROM, in which ROM is considered integral to the treatment process (no written informed consent is institutionally required for the analysis of coded data). A comprehensive protocol (Psychiatric Academic Registration Leiden database) was used, which safeguarded the anonymity of participants and ensured proper handling of the data. All participants gave permission for use of their coded data for scientific research.

## 3 INSTRUMENTS

### 3.1 Clinical global impression

The CGI is a well‐established instrument for the standardized global assessment of outcome by a rater (Guy, 1976). The scale yields two single‐item scores: one for the severity of illness (assessment of patient's current symptom severity, referred to as CGI‐S for severity) and another score for global improvement (in which a patient's current condition is compared with the baseline condition, referred to as CGI‐I for improvement). For the severity rating on the CGI‐S, raters are required to assign a patient to one of the following seven categories: 1 “Normal, not at all ill”, 2 “Borderline mentally ill”, 3 “Mildly ill”, 4 “Moderately ill”, 5 “Markedly ill”, 6 “Severely ill”, and 7 “Among the most extremely ill patients”, using “their experience with all other patients ever seen” as an explicit frame of reference. Thus, a lower score means less illness. For the improvement rating on the CGI‐I, raters assign a score according to the following scale: 1 “Very much improved”, 2 “Much improved”, 3 “Minimally improved”, 4 “No change”, 5 “Minimally worse”, 6 “Much worse”, and 7 “Very much worse”. Here, a low score means improvement, a high score means deterioration. Both CGI scores should be considered as ordinal variables, as we cannot assume that the distances among the categories of the scales are similar.

### 3.2 Brief Symptom Inventory

The BSI (Dutch version; de Beurs & Zitman, 2006; Derogatis, 1975a) is one of the most frequently used general symptom measures in mental health care. It consists of 53 items (a selection of the best‐performing items of the Symptom Checklist [SCL‐90; Derogatis, 1975b], the precursor of the BSI), each describing a “problem” (complaint or symptom). The reliability and validity of the BSI and its utility as outcome instrument are supported in many studies (for an overview, see Derogatis & Fitzpatrick, 2004). The respondent is asked to indicate “how he/she has been affected by this problem, the past week including today” on a 5‐point Likert scale ranging from 0 “none” to 4 “very much”. A total score can be calculated representing severity of general psychopathology, which was used for the present study (BSI‐TOT). A higher score means more psychopathology. BSI scores were transformed to normalized T‐scores according to *y* = 66 * (*x* + 0.01)^0.21^ and calibrated on the general population. This implies a mean score of *M* = 50 (*SD* = 10) for the general population; most patients score between *T* = 60 and *T* = 70.

## 4 GENERAL PROCEDURE

For this study, we used data collected from 2003 to 2013 at the Mental Health Care provider GGZ Rivierduinen. The collection of data is described in more detail by de Beurs et al. (2011). Here, we provide a brief description.

After a clinical intake interview by a psychiatrist and before their first treatment session, patients were invited for an assessment session in which first a semistructured diagnostic interview was administered (MINI‐plus; Sheehan et al., 1998; van Vliet and de Beurs, 2007). Next, independent assessors (research nurses or psychologists) rated the severity of the patients' symptomatology on the CGI‐S. Finally, generic (BSI) and disorder specific self‐report measures were administered by means of a computer touchscreen. Thus, when completing the CGI, raters were blind for patients' self‐reports.

Patients were reassessed every 4 to 6 months, which included the CGI improvement rating (CGI‐I). Per sampling round, the number of patients decreased with 45%, partly due to the completion of treatment, partly because of no‐show (after repeatedly being contacted) of the patient at the reassessment session. Thus, at the first assessment, *n* = 10,727 patients participated, at the second *n* = 5,900, at the third *n* = 3,245, and so forth. For *n* = 50, there was a 10th assessment. As the number of assessments varied among the patients, we censored the data at the 10th assessment and subsequently reduced the available data to the baseline, first, and last reassessment. The mean measurement interval from baseline to the first reassessment was *M* = 174 days (*SD* = 119), from baseline to the last reassessment *M* = 411 days (*SD* = 391). In particular, for the last reassessment, there was a wide range in the length of intervals from 3 months to 6 years (the maximum length of the assessment trajectory for a small number of patients).

Research nurses were thoroughly trained in administration of the MINI‐plus, the CGI and disorder specific rating scales in biweekly group sessions by rating video‐taped assessment sessions with patients and vignettes describing cases and discussing their assessment until consensus was reached.

### 4.1 Statistical analysis

First, in order to get information on the consistency/validity of the Clinical Global Impression scale, we compared change in CGI‐severity score from baseline to the first and from baseline to the last reassessment with the CGI‐improvement score by means of a measure of ordinal association (Somers' *D*; Somers, 1962). Next, we compared self‐reported severity on the total score of the BSI, with CGI‐S at three time points: baseline, first, and last reassessment with intraclass correlation coefficients (ICCs).

Furthermore, threshold values for JT_RCI_ and JT_CS_ were evaluated with receiver operating characteristics (ROC). ROC curves were calculated to investigate the sensitivity and specificity of threshold values on the BSI‐TOT T‐scores for reliable improved (JT_RCI_ > 5) with dichotomized CGI‐I as “very much” or “much improved” (1 and 2) versus “minimally improved” or a less favorable CGI‐I score (3–7) as criterion. Likewise, we evaluated the cutoff score (JT_CS_ = 55) for recovery with CGI‐S as criterion, dichotomizing CGI‐I as “very much worse” or “much worse” (6 and 7) versus minimally worse or a better outcome (1–5). Finally, we compared change scores for statistically reliable deterioration with dichotomized CGI deterioration, now dichotomizing CGI‐I as “very much worse” or “much worse” (6 and 7) versus minimally worse or a better outcome (1–5). Subsequently, outcome was categorized according to JT_RCI_, JT_CS_, and JT_RCICS_.

Subtraction of values on ordinal scales is methodologically unsound, especially when the number of levels is low and the frequency distribution of scores skewed (Wu & Leung, 2017). However, the T‐score derived from the BSI‐total score can be considered as having a genuine interval scale, which allows for subtraction of baseline and reassessment scores (BSI change scores). The association between BSI change scores and CGI‐I was investigated with Spearman's correlation coefficient rho. CGI scores are variables on an ordinal scale, and these data are analyzed with nonparametric statistical tests. Thus, we investigated the correspondence between categorizations with Somers' *D* (Somers, 1962) as measure of agreement, with the CGI‐I rating as the dependent variable.

## 5 RESULTS

### 5.1 BSI results

Mean score of patients on the BSI was *M* = 1.22 (*SD* = 0.71) at baseline, *M* = 0.88 (*SD* = 0.69) at the first reassessment, and *M* = 0.77 (*SD* = 0.68) at the last reassessment. In normalized T‐scores, this corresponds to *M* = 66.7 (*SD* = 9.3) at baseline, *M* = 60.7 (*SD* = 11.8) at the first reassessment, and *M* = 58.2 (*SD* = 12.6) at the last reassessment. Outcomes at the first reassessment categorized according to the JT approach showed that 19.6% were recovered, 29.3% were improved, 42.8% were unchanged, 6.5% were deteriorated, and 1.8% became ill. At the last available reassessment, outcomes were more favorable with 31.9% of the sample recovered, 26.9% improved, 35.5% unchanged, 5.5% deteriorated, and 2.1% became ill. As Table 1 shows, BSI‐TOT change and BSI‐TOT residual change score were significantly associated with CGI‐I scores (correlation coefficients range from *r* = .61 to *r* = .67).

**Table 1:** Associations among the outcome variables

	CGI‐I at first retest	CGI‐I at last retest
BSI change^a^	.64	.61
BSI residual change^a^	.67	.65
JT_RCI_ b	.56	.54
JT_CS_ b	.56	.54
JT_RCICS_ b	.53	.50

*Note.* All associations are statistically significant (*p* < .001).

Abbreviations: BSI, Brief Symptom Inventory; CGI‐I, clinical global impression improvement; JT_RCI_, Jacobson–Truax Reliable Change Index; JT_CS_, clinical significance; JT_RCICS_, Reliable Change Index and clinical significance combined.

a Spearman's rank correlation coefficient between BSI change scores after T‐score transformation and CGI‐I.

b Somers' *D* coefficient, CGI‐I as dependent variable.

### 5.2 CGI‐S and CGI‐I ratings

The correspondence between change in CGI‐S scores and the rating on the CGI‐I was significant: Somers' *D* = .55 for the first reassessment and Somers' *D* = .54 for the last reassessment; both *p* < .001; see Tables S1 and S2 for more detailed information. The frequency distribution of CGI‐I scores was right skewed with many more patients deemed improved than deteriorated. At the first reassessment, only 5 (0.1%) were deemed “very much deteriorated” and 57 (1.0%) “much deteriorated”; at the last reassessment, this was 7 (0.1%) and 54 (0.9%), respectively. Likewise, calculated change scores on the CGI‐S score were right skewed with only 608 cases (10.3%) with a higher severity at the first reassessment and 528 cases (9.0%) at the last reassessment. CGI‐I ratings were slightly higher than CGI‐S change scores (more observations in the lower left cells than in the upper right cells of Tables S1 and S2). At the first reassessment, patients had moved on average *M* = 0.34 BSI‐scale points (*SD* = 0.58) towards a lower severity (6.0 T‐score points or a medium effect size of ES = 0.60) and the average CGI‐I score corresponded with *M* = 3.00, (*SD* = 0.99), one scale point below the midpoint (“no change”), indicating “minimally improved.” For the last reassessment, BSI‐change was *M* = 0.45 (8.6 T‐score points), CGI‐S change was *M* = 0.95 (*SD* = 1.23) and CGI‐I was *M* = 2.78 (*SD* = 1.05), a lower mean score indicating more improvement compared with the first reassessment. Finally, more patients have convergent scores on both CGI variables than divergent scores (see Tables S1 and S2).

**Figure 1:**
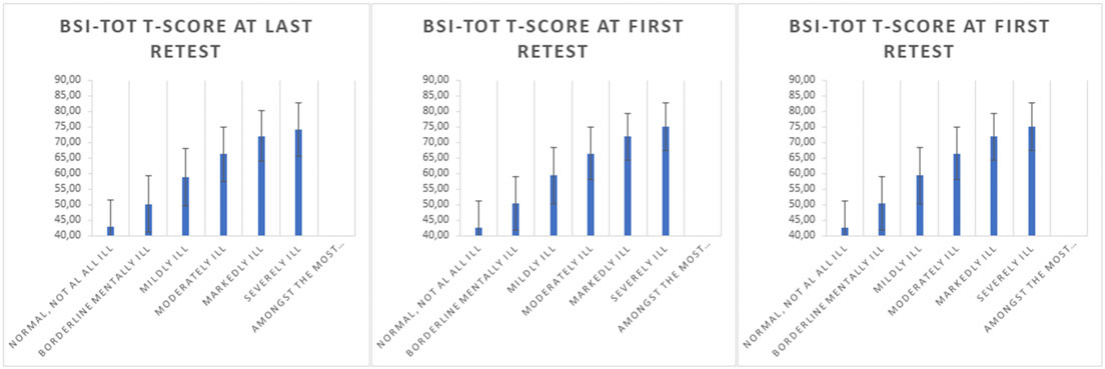
Mean scores on the BSI (normalized T‐scores) for seven severity categories at baseline, first, and last reassessment. BSI, Brief Symptom Inventory

**Figure 2:**
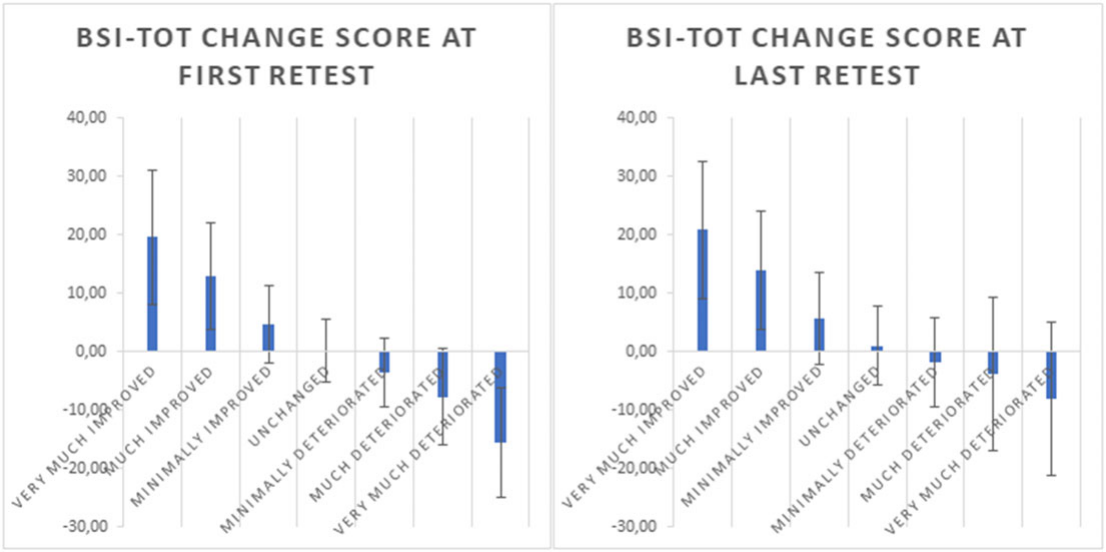
Mean change in BSI T‐scores for seven clinical global impression improvement categories at the first and last reassessment. BSI, Brief Symptom Inventory

The association between the BSI total score and CGI‐S was also substantial: ICC = .50, ICC = .68, and ICC = .70, for the baseline assessment, first, and last reassessment, respectively (all correlations *p* < .001). Figure 1 shows the mean BSI‐total score per CGI‐S category. Figure 2 shows the change in BSI‐total T‐score from baseline to first and last reassessment per CGI‐I category. All these findings support the validity of the CGI‐S and CGI‐I ratings.

### 5.3 Appropriateness of the Jacobson cutoff values

**Figure 3:**
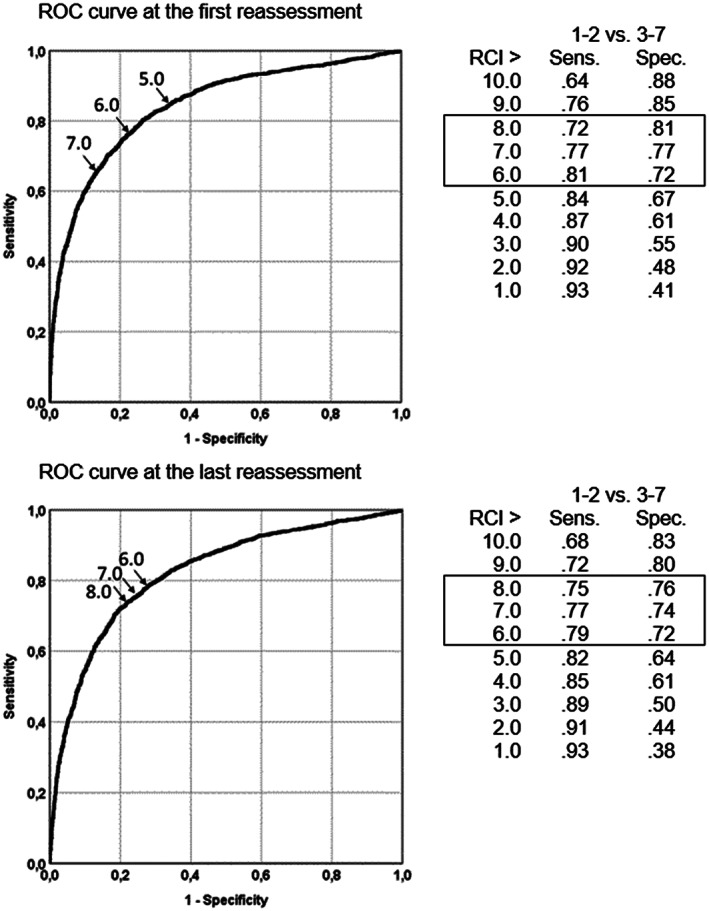
Receiver operating characteristic curves and a selection of change threshold levels (Positive Reliable Change Index values) comparing two operating characteristics: dichotomized CGI‐I (very much or much improved vs. minimally improved or worse) as the criterion of Brief Symptom Inventory total change at the first (upper) and last (lower) reassessment

Figure 3 shows the ROC curves for the first and the last reassessment and several coordinates of the curve (Reliable Change Index threshold values) for change in BSI T‐scores. The area under the ROC curve for all possible BSI change scores at the first reassessment was AUC = .84 (95% CI [.83, .85]) and at the last reassessment AUC = .82 (95% CI [.81, .83]). The results suggest JT_RCI_ > 6.0 as optimum threshold value. With RCI > 5 at the last reassessment, 84% of changed cases are deemed very much or much improved, and 67% of unchanged cases are deemed minimally improved or less. Results of the last reassessment are similar.

**Figure 4:**
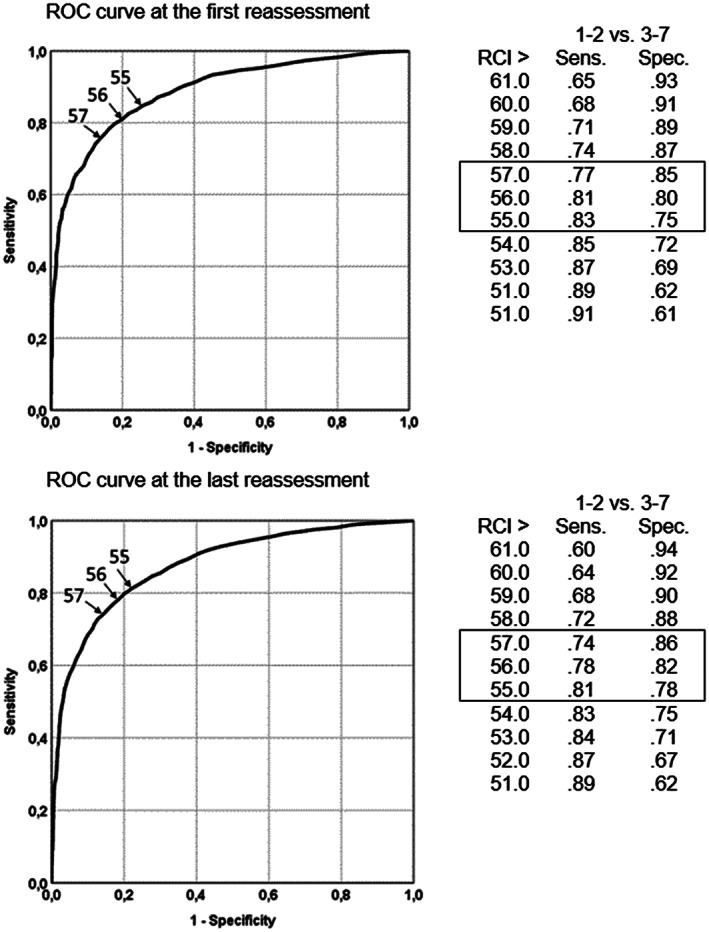
Receiver operating characteristic curves and a selection of reassessment T‐score threshold levels (JTCS cutoff values) comparing two operating characteristics: dichotomized CGI‐S (not at all or borderline mentally ill vs. mildly to severely ill) as the criterion of Brief Symptom Inventory total T‐score at the first (upper) and last (lower) reassessment

Figure 4 presents the ROC curves and sensitivity and specificity indices for threshold values for JT_CS_ on the BSI (T‐score). The area under the ROC curve was AUC = .82 (95% CI [.81, .83]) and AUC = .89 (95% CI [.88, .90]) for the first and the last reassessment, respectively. The findings suggest JT_CS_ = 56.0 as optimum threshold value for clinical significance.

**Figure 5:**
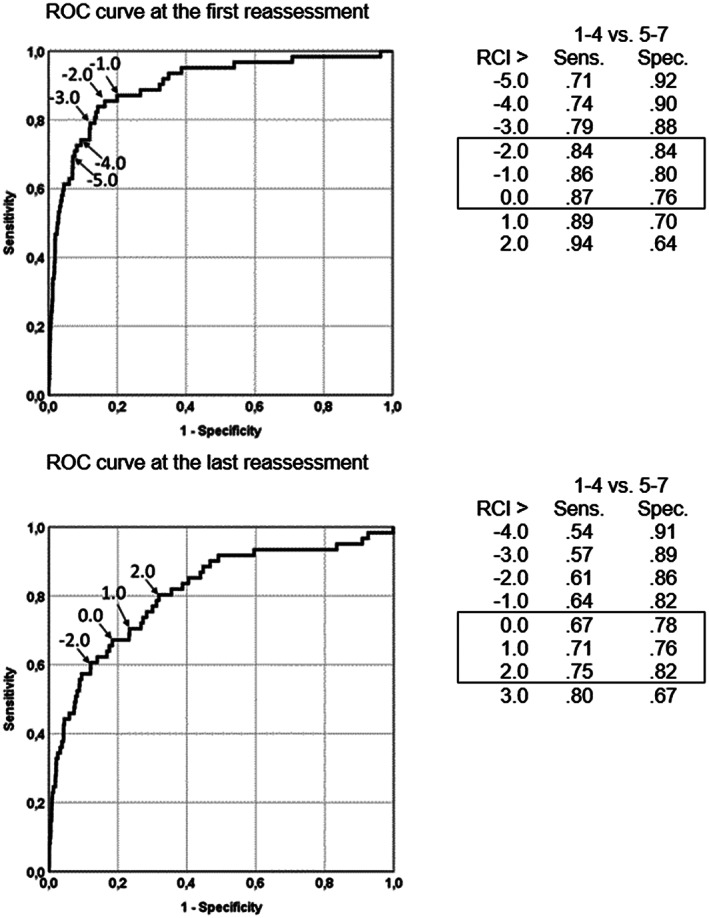
Receiver operating characteristic curves and a selection of change threshold levels (Negative Reliable Change Index values) comparing two operating characteristics: dichotomized CGI‐I (very much or much deteriorated vs. unchanged or better) as the criterion of Brief Symptom Inventory total change at the first (upper) and last (lower) reassessment

Finally, Figure 5 presents two ROC curves for the cutoff for reliable deterioration: AUC = .90 (95% CI [.86, .94]) for the first reassessment and AUC = .81 (95% CI [.75, .87]) for the last reassessment. The optimum threshold value according to the ROC curve differs considerably from JT_RCI_ < −5 in both curves, probably due to the few patients that were considered deteriorated on the CGI‐I.

### 5.4 Concordance between JT and CGI

The agreement between outcome according to the JT approach and according to the CGI‐I was statistically significant (all associations *p* < .05) and also substantial, as indicated by the index of association for ordered variables (Somers' *D*, between JT_RCI_, JT_CS_, and JT_RCICS_ on the one hand and CGI‐I on the other hand; see Table 1). Tables 2 and 3 present the numbers of patients in convergent and divergent categories for JT_RCI_ and CGI‐I (with CGI‐I reduced to three categories for a clearer presentation of the findings). These results indicate that the CGI‐I gave a somewhat more conservative estimate of treatment outcome than JT_RCI_: the largest off‐diagonal group (*n* = 1,360) had “minimal or no change” according to the CGI‐I but were reliably improved according to JT_RCI_ (see Table 2). Similarly, at the last reassessment, almost all reliably improved patients were also deemed improved according to the CGI‐I, but also 1,301 of the reliably improved cases were deemed “minimal or not changed” (see Table 3). We tested higher threshold values for RCI (6, 7, and 8), but this did increase the association between JT_RCI_ and CGI only marginally (e.g., with JT_RCI_ = 7, Somers' *D* would increase from *D* = .53 to *D* = .57).

**Table 2:** Number of cases (row %) categorized according to the Jacobson–Truax approach and according to CGI improvement (reduced to three or five categories) at the first reassessment (large off‐diagonal disagreeing categories in bold typeface)

	Global clinical impression improvement (in three categories)					
JT_RCI_	(Very) much improved		Minimal or no change	(Very) much deteriorated		Total (column %)
Reliably improved	1,525 (52.8)		**1**,**360** (**47.1**)	3 (0.1)		2,888 (48.9)
Unchanged	251 (9.9)		2,257 (89.5)	15 (0.6)		2,532 (42.8)
Reliably deteriorated	38 (7.8)		407 (83.2)	44 (9.0)		489 (8.3)
Total	1,814 (30.7)		4,024 (68.2)	62 (1.1)		5,900 (100)
JT_CS_	(Very) much improved		Minimal or no change	(Very) much deteriorated		Total (column %)
Recovered	881 (71.0)		**358** (**28.9**)	1 (0.0)		1,240 (21.0)
Unchanged	**918** (**20.3**)		3,556 (78.6)	49 (1.1)		4,523 (76.7)
Became ill	15 (10.9)		110 (80.3)	12 (8.8)		137 (2.3)
Total	1,814 (30.7)		4,024 (68.2)	62 (1.1)		5,900 (100)
	Global clinical impression improvement (in five categories)					
JT_RCICS_	Very much improved	Much improved	Minimal or no change	Much deteriorated	Very much deteriorated	
Recovered	173 (15.0)	**683** (**59.0**)	**300** (**25.9**)	1 (0.1)	0 (0.0)	1157 (19.6)
Reliably improved	79 (4.6)	590 (34.1)	**1**,**060** (**61.2**)	2 (0.1)	0 (0.0)	1731 (29.3)
Unchanged	19 (0.8)	232 (9.2)	2,257 (89.5)	15 (0.6)	0 (0.0)	2532 (42.8)
Reliably deteriorated	3 (0.8)	25 (6.6)	321 (84.3)	28 (7.3)	4 (1.1)	381 (6.5)
Became ill	2 (1.9)	8 (7.4)	86 (79.6)	11 (10.2)	1 (0.7)	108 (1.8)
Total	276 (4.7)	1538 (26.1)	4,024 (68.2)	57 (1.0)	5 (0.1)	5900 (100)

Abbreviations: JT_RCI_, Jacobson–Truax Reliable Change Index; JT_CS_, clinical significance; JT_RCICS_, Reliable Change Index and clinical significance combined.

**Table 3:** Number of cases (row %) categorized according to the Jacobson–Truax approach and according to CGI improvement (reduced to three or five categories) at the last reassessment (large off‐diagonal disagreeing categories in bold typeface)

	Global clinical impression improvement (in three categories)					
JT_RCI_	(Very) much improved		Minimal or no change	(Very) much deteriorated		Total (column %)
Reliably improved	2,161 (62.3)		**1**,**301** (**37.5**)	9 (0.3)		3471 (58.8)
Unchanged	322 (16.3)		1,633 (82.5)	24 (1.2)		1,979 (33.5)
Reliably deteriorated	60 (13.3)		362 (80.4)	28 (6.2)		450 (7.6)
Total	2,543 (43.1)		3,296 (55.9)	61 (1.0)		5,900 (100)
JT_CS_	(Very) much improved		Minimally or no changed	(Very) much deteriorated		Total (column %)
Recovered	1,381 (77.2)		**406** (**22.7**)	3 (0.2)		1,790 (30.3)
Unchanged	**1,136** (**28.6**)		2,792 (70.2)	49 (1.2)		3,977 (63.9)
Became ill	26 (19.5)		98 (73.7)	9 (6.8)		133 (2.3)
Total	2,543 (43.1)		3,296 (55.9)	61 (1.0)		5,900 (100)
	Global clinical impression improvement (in five categories)					
JT_RCICS_	Very much improved	Much improved	Minimally or no changed	Much deteriorated	Very much deteriorated	
Recovered	366 (21.2)	**987** (**57.2**)	**370** (**21.4**)	3 (0.2)	0 (0.0)	1,726 (31.9)
Reliably improved	109 (6.2)	699 (40.1)	**931** (**53.4**)	5 (0.3)	1 (0.1)	1,745 (26.9)
Unchanged	31 (1.6)	291 (14.7)	1,633 (82.5)	21 (1.1)	3 (0.2)	1,979 (33.5)
Reliably deteriorated	5 (1.4)	39 (11.1)	289 (82.1)	17 (4.8)	2 (0.6)	352 (5.5)
Became ill	3 (3.1)	13 (13.3)	73 (74.5)	8 (8.2)	1 (1.0)	98 (2.1)
Total	514 (4.7)	2,029 (26.1)	3,269 (68.2)	54 (0.9)	7 (0.1)	5,900 (100)

Abbreviations: JT_RCI_, Jacobson–Truax Reliable Change Index; JT_CS_, clinical significance; JT_RCICS_, Reliable Change Index and clinical significance combined.

In contrast, JT_CS_ is somewhat more conservative than CGI‐I, with more cases in the opposite categories “unchanged” but “(very) much improved” (*n* = 918 and *n* = 1,136 at the first and last reassessment) than in the opposite categories “recovered” but “minimal or no change” (*n* = 358 and *n* = 406). Finally, comparing the outcome according to the full JT_RCICS_ categorization with CGI‐I revealed a pattern similar to what was found for JT_RCI_: the CGI‐I being a somewhat more conservative estimate of treatment success than the JT_RCICS_ index, due to recovered and reliable improved patients according to JT_RCICS_, who are merely improved or not changed according to the CGI‐I. Changing the cutoff values for JT_RCI_ or JT_CS_ did not improve the association between CGI‐I and JT_RCICS_.

## 6 DISCUSSION

### 6.1 Overview of the main findings

The findings of the present study revealed that CGI scores were more strongly associated with the continuous BSI‐TOT change and residual change scores than with JT indices. As has been argued before (Fedorov, Mannino, & Zhang, 2009; Markon, Chmielewski, & Miller, 2011), information is lost when converting continuous scores to JT categories, diminishing the association of the latter with CGI‐I.

However, the categorization of patients according to the JT approach and CGI ratings by an independent evaluator corresponded still quite well, both at the first and at the last reassessment, supporting the validity of the JT categorization and the appropriateness of the proposed threshold values for statistically reliable and clinical change. The latter was also demonstrated with the ROC analyses, which show high sensitivity and specificity for the threshold values. An exception is the sensitivity and specificity of the threshold for reliable deterioration. Here, optimal sensitivity and specificity are not at −5 but at a positive change score of 1. The low base rate of deteriorated cases on the CGI‐I criterion plays a role here, as this limits the sensitivity to detect deteriorated cases through the BSI change score (Meehl & Rosen, 1955).

An advantage of the JT approach is that it reveals information otherwise missed. For instance, remarkably few patients are categorized in the worst outcome group of those becoming ill (only 1.8% at the first reassessment and 2.1% at the last reassessment). This low number may actually be an underestimation due to selection bias, as patients who deteriorate are more likely to drop out from treatment and/or may be less inclined to comply with a reassessment appointment. Consequently, they are probably not fully represented in the current dataset.

### 6.2 Validity of the CGI

The CGI has been criticized on semantical, logical, and psychometric grounds (Beneke & Rasmus, 1992), but in practice, this instrument performs well (Berk et al., 2008; Leucht & Engel, 2005) as evidenced by high associations between CGI‐S change scores and CGI‐I scores and by sufficient concurrent validity with other outcome measures (Haro et al., 2003; Leon et al., 1993). In our study, improvement according to CGI‐I was also significantly associated with change in CGI‐Severity scores (Somers' *D* = .55 and .54 for the first and last reassessment). Few patients who were deemed improved were deteriorated in the preceding interval according to their CGI‐S scores (*n* = 192), and even less who had worsened were improved according to CGI‐S scores (*n* = 30). Comparable numbers were found at the last reassessment (see Tables S1 and S2 with the categories in bold typeface). Similar findings for the validity of the CGI are reported by Berk et al. (2008; e.g., correlation between change in CGI‐S and CGI‐I: *r* = .71). These findings boost our confidence in the suitability of the CGI‐I as criterion to evaluate JT threshold values.

### 6.3 Appropriateness of the threshold values for JT_RCI_, JT_CS_, and JT_RCICS_

The comparisons of the categorizations according to JT and CGI‐I revealed substantial concordance between both approaches, certainly if one considers that some discordance is to be expected between patients' self‐reports and ratings by independent evaluators. Independent evaluators have only limited access to relevant clinical details and have to base their rating on information provided by the patient at the assessment session. The CGI rating may get biased towards the positive when patients present a too positive picture of the treatment gains, the Hello‐Goodbye effect (Hathaway, 1948). Also, independent evaluators themselves may be inclined to view the outcome of treatment more favorably than actually was achieved, as sound judgement can be clouded by wishful thinking or other biases (Kahneman, 2011),

In addition, discrepancy between raters' evaluations and patient self‐reports may result from threats to the validity of self‐report data, such as response shift bias in patients' self‐reports. This refers to changes in the meaning of one's self‐evaluation, which results from changes in internal standards, values, or conceptualization of disease symptoms (Sprangers & Schwartz, 1999). Basically, patients learn more about their condition and symptoms (e.g., by psychoeducation), which potentially affects their reassessment scores towards reporting more symptoms. This diminishes baseline‐to‐reassessment change scores, obscures true change, and consequently may diminish the association between self‐reports and observer ratings.

The categorization based on CGI‐I yielded a somewhat more conservative estimate of treatment outcome compared with the categorization JT_RCICS_. Many patients meeting JT_RCICS_ for improvement or recovery were rated as not changed or minimally improved. This is mainly explained by disagreement between the Reliable Change Index JT_RCI_ and CGI‐I. A higher threshold value for JT_RCI_ could be applied, such as 6 or 7 (as suggested by the present ROC analyses), but this did not yield a higher association with CGI‐I. Alternatively, an outcome measure with higher measurement precision (e.g., disorder‐specific measure, such as the Beck Depression Inventory; Beck & Steer, 1987) may yield results that are more concordant with the CGI rating.

### 6.4 Clinical application

Currently, for a proper interpretation of test results, detailed knowledge of a measurement instrument is required, as each instrument has its own scale and range of scores. This complicates comparison of scores across patients unnecessarily. There is a growing interest in T‐scores as a common metric for health assessment questionnaires and crosswalk tables are published for the transformation of scores (Choi, Schalet, Cook, & Cella, 2014; Rose & Devine, 2014; Schalet, Cook, Choi, & Cella, 2014; Wahl et al., 2014). The present findings support the utility of two cutoff values for meaningful change: A 5‐point change in T‐score implies a change beyond the measurement error of the BSI and likely represents a true change in severity of psychopathology; a T‐score of 55 or less implies that it is more likely that the respondent stems from the functional population than the dysfunctional population. These straightforward cutoffs ease the interpretation of measurement results and may stimulate professionals in mental health care to make better use of ROM information during treatment (Fortney et al., 2017) and inform and involve patients better in a shared decision‐making context (Simon, Wills, & Härter, 2009).

### 6.5 Strengths and limitations

Strengths of the study are that the appropriateness of cutoff values for reliable change and clinical significance were empirically tested with ROC analyses in a sizable sample. Correspondence between JT categories and CGI was assessed from different angles, and all analyses were replicated within the dataset using the longer interval of maximum 10 assessments.

A further strength of the study is that CGI‐I was derived from independent evaluators and not from therapists. Therapist may have a too positive outlook on the results achieved with treatment (Hatfield, McCullough, Frantz, & Krieger, 2010; Lilienfeld, Ritschel, Lynn, Cautin, & Latzman, 2014; Walfish, McAlister, O'Donnell, & Lambert, 2012). Indeed, research shows that independent evaluators appear to yield a more conservative estimate of treatment gains (Fox & Warner, 2017). However, Lewin, Peris, De Nadai, McCracken, and Piacentini (2012) found similar ratings from independent evaluators and therapists, but both rated treatment gains more conservatively compared with children (the subjects in their psychotherapy trial) themselves and their parents. More research is needed to evaluate the extent of bias in CGI ratings from different sources.

The present study used longitudinal data from a patient sample with common mental disorders who were treated in everyday clinical practice and participated in an observational study, enhancing the generalizability of the findings. However, data collection in real life circumstances introduces more noise in the data, resulting from varied reassessment intervals and substantial (and potentially selective) loss of data. In particular, the latter is a point of concern as this may have influenced the outcome data in opposite ways. On the one hand, selection bias may have inflated estimated treatment outcome, as unsuccessfully treated patients are more likely to decline participation in reassessments. On the other hand, there were also many patients who continued treatment and continued to improve but declined further reassessments. For these patients, no endpoint assessment was available, which may have deflated the overall outcome estimates. This precludes the use of the present results for definitive statements about what is achieved with mental health care in everyday clinical practice. However, for the purpose of comparing two indicators of treatment outcome—JT and CGI‐I—the present data are still quite suitable.

Future research into the appropriateness of the JT criteria for categorization of treatment outcome may focus on other, more objective indicators of treatment outcome, such as ensuing treatment or readmission to mental health care later on. In particular, long‐term follow‐up data could provide relevant information on the aptness of the JT categorization with the proposed cutoff values.

## 7 CONCLUSION

The results revealed support for the validity of the JT method and the proposed cutoff values for reliable change (RCI > 5 and CS = 55) associated with the JT method. The JT method appeared somewhat more optimistic about what had been achieved compared with the CGI‐improvement ratings. There was less support for the distinction between unchanged from deteriorated patients among patients with an unfavorable treatment outcome. The JT method translates pretest‐to‐posttest change scores into clinically relevant outcome categories with immediate appeal to the clinician. The categories correspond reasonably well with improvement ratings by an independent rater.

## DECLARATION OF INTEREST STATEMENT

The author(s) declare that they have no competing interests.

## AUTHORS CONTRIBUTIONS

E. de. B. contributed to conception and design of the study, conducted the statistical analysis, and wrote the manuscript. I. V. E. C. participated in the design of the study, coordinated the study and data acquisition, and revised the manuscript. A. M. v. H. has been involved in revising the manuscript. All authors read and approved the final manuscript.

## Supporting information

Table S1. Change in CGI severity scores by CGI improvement scores at 1^st^ reassessment
Table S2. Change in CGI severity scores by CGI improvement scores at the last reassessment

## ENDNOTES

1 Calculated as $\mathrm{CS}=\frac{\left({\mathrm{SD}}_{1}*M_{2}+{\mathrm{SD}}_{2}*M_{1}\;\right)}{M_{1}+M_{2}}$.

2 The values are appropriate when raw scores on outcome measures are transformed to T‐scores, which are calibrated on the general population.
