# Supplementary material for: Approaches to denote treatment outcome: Clinical significance and clinical global impression compared
Source: Int J Methods Psychiatr Res. 2019 Oct 9;28(4):e1797. doi: 10.1002/mpr.1797 (PMC7649961; doi:10.1002/mpr.1797)
Supplement: Supplementary file 1 — Table S1. Change in CGI severity scores by CGI improvement scores at 1st reassessment Table S2. Change in CGI severity scores by CGI improvement scores at the last reassessment [file MPR-28-e1797-s001.docx]

Supplementary materials

Table S1. Change in CGI severity scores by CGI improvement scores at 1^st^ reassessment

|  |  | CGI-improvement | | | | | | |  |
| --- | --- | --- | --- | --- | --- | --- | --- | --- | --- |
| CGI-S change |  | Very much improved | Much improved | Minimally improved | No change | Minimally deteriorated | Much deteriorated | Very much deteriorated | Total (Column %) |
| Improvement { | >2 | 119 (36.7) | 183 (56.5) | 17 (5.2) | 4 (1.2) | 1 (0.3) | 0 (0.0) | 0 (0.0) | 324 (5.5) |
|  | 2 | 96 (11.8) | 516 (63.2) | 180 (22.0) | 22 (2.7) | 3 (0.4) | 0 (0.0) | 0 (0.0) | 817 (13.8) |
|  | 1 | 45 (2.4) | 579 (31.1) | 1010 (54.3) | 199 (10.7) | 22 (1.2) | 4 (0.2) | 0 (0.0) | 1859 (31.5) |
| Identical score | 0 | 16 (0.7) | 242 (10.6) | 1061 (46.3) | 819 (35.7) | 143 (6.2) | 11 (0.5) | 0 (0.0) | 2292 (38.8) |
| Deterioration { | 1 | 0 (0.0) | 15 (2.9) | 160 (30.7) | 204 (39.1) | 117 (22.4) | 22 (4.2) | 4 (0.8) | 522 (8.8) |
|  | 2 | 0 0.0() | 3 (3.8) | 12 (15.4) | 22 (28.2) | 23 (29.5) | 17 (21.8) | 1 (1.3) | 78 (1.3) |
|  | <2 | 0 (0.0) | 0 (0.0) | 2 (25.0) | 2 (25.0) | 1 (12.5) | 3 (37.5) | 0 (0.0) | 8 (0.1) |
| Total |  | 276 (4.7) | 1538 (21.626.1) | 2442 (41.4) | 1272 (21.6) | 310 (5.3) | 57 (1.0) | 5 (0.1) | 5900 (100) |

Table S2. Change in CGI severity scores by CGI improvement scores at the last reassessment

|  |  | CGI-improvement | | | | | | |  |
| --- | --- | --- | --- | --- | --- | --- | --- | --- | --- |
| CGI-S change |  | Very much improved | Much improved | Minimally improved | No change | Minimally deteriorated | Much deteriorated | Very much deteriorated | Total (Column %) |
| Improvement { | >2 | 262 (38.8) | 357 (52.8) | 47 (7.0) | 8 (1.2) | 2 (0.3) | 0 (0.0) | 0 (0.0) | 676 (11.5) |
|  | 2 | 156 (14.1) | 701 (63.3) | 214 (19.3) | 31 (2.8) | 5 (0.5) | 1 (0.1) | 0 (0.0) | 1108 (18.8) |
|  | 1 | 80 (4.4) | 669 (36.7) | 808 (44.3) | 213 (11.7) | 43 (2.4) | 7 (0.4) | 2 (0.1) | 1822 (30.9) |
| Identical score | 0 | 15 (0.8) | 267 (15.1) | 755 (42.8) | 579 (32.8) | 135 (7.6) | 14 (0.8) | 1 (0.1) | 1766 (29.9) |
| Deterioration { | 1 | 1 (0.2) | 29 (6.5) | 142 (32.1) | 169 (38.1) | 81 (18.3) | 18 (4.1) | 3 (0.7) | 443 (7.5) |
|  | 2 | 0 (0.0) | 6 (7.9) | 21 (27.6) | 19 (25.0) | 17 (22.4) | 12 (15.8) | 1 (1.3) | 76 (1.3) |
|  | <2 | 0 (0.0) | 0 (0.0) | 1 (11.1) | 4 (44.4) | 2 (22.2) | 2 (22.2) | 0 (0.0) | 9 (0.2) |
| Total |  | 514 (8.7) | 2029 (34.4) | 1988 (33.7) | 1023 (17.3) | 285 (4.8) | 54 (0.9) | 7 (0.1) | 5900 (100) |
